# Factors associated with high-grade cervical dysplasia in colposcopy patients: the primary role of isolated HPV 16 positivity

**DOI:** 10.3389/fmed.2026.1937610

**Published:** 2026-10-01

**Authors:** Alihan Tigli, Yakup Baykus, Rulin Deniz, Oguzhan Karakoc, Guzide Ece Akinci, Nazli Sener, Yasemin Ercan Degirmenci, Erdem Gurkan, Engin Korkmazer, Seyda Yavuzkir, Ahmet Yilmaz, Berrak Yildirim Aksakal, Sermin Kilic, Suleyman Aydin

**Affiliations:** 1 Department of Obstetrics and Gynaecology, Faculty of Medicine, Bandırma Onyedi Eylül University, Bandırma, Türkiye; 2 Gynecology and Obstetrics Clinic, Bandırma Training and Research Hospital, Bandırma, Türkiye; 3 Gynecology and Obstetrics Clinic, Şehit Prof. Dr. İlhan Varank Sancaktepe Training and Research Hospital, Istanbul, Türkiye; 4 Obstetrics and Gynecology Private Practice, Bursa, Türkiye; 5 Department of Obstetrics and Gynaecology, Faculty of Medicine, Fırat University, Elazığ, Türkiye; 6 Department of Medical Biochemistry, Faculty of Medicine, Fırat University, Elazığ, Türkiye; 7 Department of Medical Education, Faculty of Medicine, Fırat University, Elazığ, Türkiye; 8 Department of Obstetrics and Gynecology, Elazığ Fethi Sekin City Hospital, Elazığ, Türkiye

**Keywords:** age, cervical dysplasia, cervical intraepithelial neoplasia, CIN, colposcopy, HPV

## Abstract

**Background:**

Cervical cancer is the fourth most common cancer and a leading cause of cancer-related fatalities among women globally. Implementing personalized follow-up strategies is crucial for effective prevention of cervical cancer. The present investigation sought to delineate risk factors associated with the development of cervical intraepithelial neoplasia (CIN) in patients undergoing colposcopy through an analysis of colposcopy indications and histopathological outcomes.

**Methods:**

This retrospective study included 394 patients aged 30–65 years who underwent colposcopy at Bandırma Onyedi Eylül University Faculty of Medicine Hospital between June 2022 and January 2025 due to high-risk Human Papillomavirus (HR-HPV) positivity, abnormal cervical cytology, or persistent clinical suspicion. The indications for colposcopy, histopathology results, and demographic characteristics such as age and smoking status were analyzed.

**Results:**

The mean age of the patients was 44.5 ± 10.9 years. The primary indication for colposcopy was observed to be isolated HPV 16 positivity, which accounted for 28% (*n* = 112) of cases. Histopathological evaluation showed no cervical dysplasia in 47.3% (*n* = 186), CIN I in 34.5% (*n* = 136), CIN II–III in 17.7% (*n* = 70), and cervical carcinoma in 0.5% (*n* = 2). Isolated HPV 16 positivity remained a significant independent predictor of CIN II–III after adjustment for age and smoking status (Adjusted Odds Ratio (AOR) = 2.388, 95% Confidence Interval (CI): 1.387–4.112, *p* < 0.05).

**Conclusions:**

Isolated HPV 16 positivity was independently associated with an approximately 2.4-fold increase in the odds of CIN II–III after adjustment for age and smoking status. This finding reinforces the central role of HPV 16 in identifying women at higher risk for high-grade cervical dysplasia and supports its prioritization in risk-based colposcopy and follow-up strategies.

## 1 Introduction

Cervical cancer is one of the most prevalent cancers affecting women worldwide and threatens the lives of millions each year. According to World Health Organization (WHO) data, it is a leading cause of cancer-related mortality among women. Regular and systematic screening programs are crucial for the prevention and early detection of cervical cancer. Among screening methods, testing for oncogenic subtypes of Human Papillomavirus (HPV) is widely used alongside cervical cytology (Pap smear). HPV infection is recognized as a primary etiological factor in the development of cervical cancer. In particular, high-risk types, especially HPV 16 and HPV 18, play a major role in the pathogenesis of cervical dysplasia and invasive cervical carcinoma (1).

In Türkiye, the national population-based cervical cancer screening program primarily uses HPV-DNA testing. Women aged 30–65 years are offered free HPV-DNA screening at 5-year intervals through primary healthcare services. Cervical cytology is not routinely used as a co-test within the population-based screening program, although cytological assessment may be performed as part of further clinical evaluation when indicated. HPV vaccination is currently not mandatory and is not included in the routine national immunization schedule; it is available on a voluntary, self-funded basis (through out-of-pocket payment)*.* This screening strategy enables the identification of women at increased risk for cervical dysplasia and supports timely diagnostic evaluation and intervention (2).

Colposcopy serves as a critical diagnostic procedure performed after abnormal screening results and is an essential tool for detecting and grading cervical dysplasia and cancer. Biopsy samples taken during colposcopic examination help determine the severity of cervical dysplasia through histopathological analysis. Colposcopic biopsy is a vital step in confirming the diagnosis of cervical dysplasia and guiding appropriate treatment interventions. In this context, analyzing colposcopy findings offers valuable insights into the causes and prevalence of cervical dysplasia (3).

Although significant progress has been made in preventing and treating cervical cancer in recent years, there are still some challenges in the screening and treatment processes of cervical dysplasia and cancer in Turkey. There is a continued need to enhance public awareness regarding HPV, encourage participation in national screening programs, and improve access to healthcare services for early diagnosis and treatment. Additionally, considering regional variation in epidemiology and healthcare access is essential for designing effective and equitable screening strategies.

The present study seeks the evaluation of the indications, results, and histopathological findings of colposcopy procedures conducted at the Gynaecology Clinic of Bandırma Onyedi Eylül University Faculty of Medicine Hospital. Through the comparison of these results with the existing national and international literature, it is suggested that this investigation may contribute to the advancement of cervical dysplasia screening strategies and the establishment of a foundation for more effective approaches regarding the early diagnosis and clinical management of cervical dysplasia and carcinoma.

## 2 Materials and methods

### 2.1 Study design, setting, and participants

The study cohort comprised female patients who underwent colposcopy at the Gynaecology Clinic of Bandırma Onyedi Eylül University Faculty of Medicine Hospital between June 2022 and January 2025. A total of 394 patients aged 30–65 years were enrolled. Inclusion criteria comprised (1) a positive test for at least one HR-HPV type using the Bio-Rad Cycling Fast Extension Polymerase Chain Reaction (CFX-PCR) method (including concurrent β-globin internal control for sample adequacy) (2), abnormal cervical cytology based on the 2014 Bethesda System, and (3) clinical suspicion of persistent cervical pathology necessitating colposcopy. All cervical cytology samples were collected by trained clinicians following standard clinical procedure.

Exclusion criteria included (1) cases involving current pregnancy (2), a history of treatment for cervical cancer or cervical preinvasive lesions (3), previous hysterectomy (4), radiotherapy or chemotherapy (5), HPV vaccination or immunodeficiency, and (6) cases with incomplete records (Figure 1).

**Figure 1:**
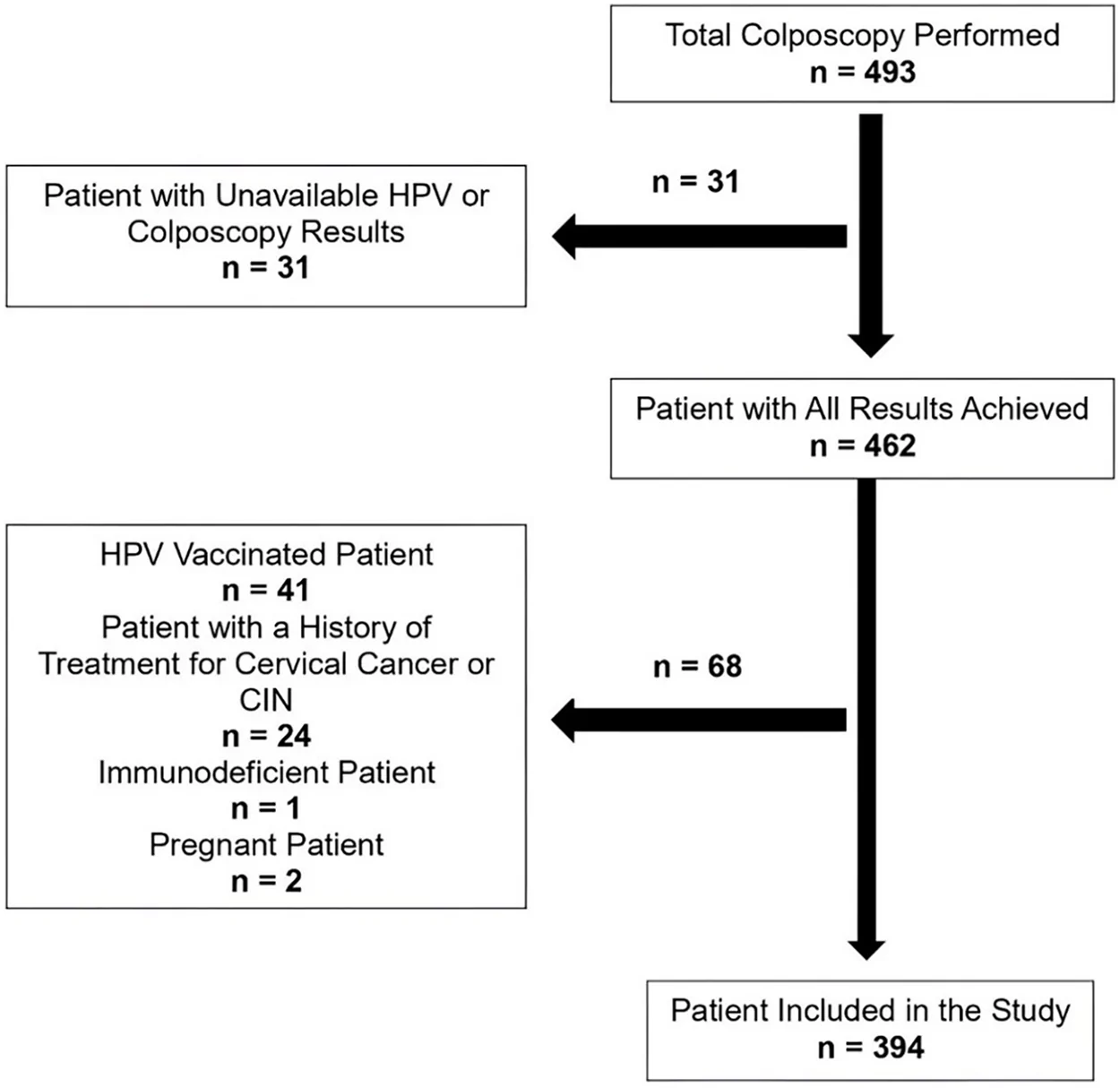
Flow chart of patients undergoing colposcopy. HPV, Human Papillomavirus; CIN, cervical intraepithelial neoplasia.

At our center, women testing positive for HPV 16 and/or HPV 18 are referred directly for colposcopy, irrespective of reflex cytology findings. In patients with both HR-HPV positivity and abnormal cervical cytology, classification was based primarily on HPV genotype. Accordingly, co-positive women were assigned to the corresponding HPV genotype group (Groups 1–4), whereas Group 6 included only HPV-negative women with abnormal cervical cytology.

The cases were divided into six groups according to the indications for colposcopy:

Group 1: Patients positive for HPV 16 only.
Group 2: Patients positive for HPV 18 only.
Group 3: Patients positive for both HPV 16 and 18.
Group 4: Patients negative for HPV 16 and 18 but positive for at least one other HPV type.
Group 5: Patients negative for HPV, with normal smear results and only clinical suspicion on vaginal examination (which may be defined by the presence of cervical ectropion, abnormal cervical appearance, contact bleeding, or atypical discharge).
Group 6: Patients negative for HPV but with abnormal findings on cervical cytology (Low-grade Squamous Intraepithelial Lesion (LSIL), High-grade Squamous Intraepithelial Lesion (HSIL), Atypical Squamous Cells – cannot exclude HSIL (ASC-H), Atypical Glandular Cells (AGC)).

Experienced gynecologists conducted colposcopic examinations using a binocular colposcope equipped with green and blue filters and up to 40× magnification, in accordance with internationally accepted guidelines, specifically the American Society for Colposcopy and Cervical Pathology (ASCCP) recommendations. Biopsy specimens were collected in a clockwise order. The highest grade reported in the histopathology results was considered the final diagnosis for each patient. Histopathological diagnoses were extracted directly from the original pathology reports, which used the conventional three-tier CIN terminology. Because this was a retrospective study and ancillary p16 immunohistochemical assessment was not uniformly available for all lesions requiring LAST-based adjudication, retrospective reclassification according to the two-tier LAST system was not performed. For analytical purposes, CIN II and CIN III were combined as a high-grade disease category, broadly corresponding to HSIL. In the context of this study, the term “cervical dysplasia” is utilized as an overarching category encompassing all pathological lesions (CIN I, CIN II–III, and cervical carcinoma) to distinguish them from non-dysplastic outcomes.

### 2.2 Study design

This retrospective, cross-sectional study involved a review of patient files and electronic records, and the reporting was conducted in accordance with the Strengthening the Reporting of Observational Studies in Epidemiology (STROBE) statement.

### 2.3 Data collection method

Demographic data, colposcopy indications, and histopathology results were retrospectively extracted via the hospital automation system and archive records. Parity data were not consistently available in the retrospective medical records and therefore could not be included in the statistical analyses.

### 2.4 Statistical analysis

The analyses were conducted using SPSS version 23.0 (IBM Corp., Armonk, NY, USA). Descriptive statistics, including frequency, percentage, mean, and standard deviation, were used to summarize the findings. The Pearson chi-square test was used to compare histopathological outcomes according to colposcopy indications, age, and smoking status when expected cell counts were adequate, whereas Fisher's exact test was applied when expected frequencies were small, particularly in analyses involving limited subgroups such as HPV 16/18 co-positivity. For proportions, 95% confidence intervals (CIs) were calculated. The association between colposcopy indications and histopathological results was further elucidated through the application of multivariate logistic regression analysis. In all models, colposcopy indications (Isolated HPV 16, Isolated HPV 18, HPV 16 and HPV 18 co-positivity, Other HR-HPV, Clinical suspicion, and Abnormal cervical cytology) were included as independent variables, while histopathological results (cervical dysplasia, CIN I, and CIN II–III) served as dependent variables. All categorical variables were coded as dummy variables (0 = negative, 1 = positive), with the negative status serving as the reference group for each specific indication. In addition to the aforementioned dependent and independent variables, age (continuous) and smoking status (No = 0, Yes = 1) were included as covariates in Models 2, 3, and 4. Age was retained as a continuous variable in all multivariable logistic regression models to avoid loss of information. The dichotomization of age into ≤44 and ≥45 years was used only for descriptive subgroup comparisons presented in Table 3. These variables were strategically selected to adjust for their well-established confounding effects on cervical dysplasia progression, as widely documented in the literature. For descriptive age-based comparisons in Table 3, patients were categorized as ≤44 and ≥45 years based on the mean age of the study population (44.5 years); however, this categorization was not used in the multivariable regression analyses. A *p*-value < 0.05 was considered statistically significant for all statistical tests.

### 2.5 Ethical approval

The study was conducted in accordance with the Declaration of Helsinki and was approved by the Bandırma Onyedi Eylül University Health Sciences Non-Interventional Research Ethics Committee (10 April 2025; Decision No. 2025-33). The requirement for informed consent was waived because of the retrospective design and the use of anonymized patient data.

## 3 Results

Upon examination of data from 394 patients, findings indicated that participant ages ranged from 30 to 65 years (mean 44.5 ± 10.9 years), while the prevalence of smoking was 59%.

### 3.1 Indications for colposcopy

The most prevalent indication for colposcopy was isolated HPV 16 positivity (28%; *n* = 112), followed by abnormal cervical cytology (24%; *n* = 93) and clinical suspicion (21%; *n* = 82); conversely, the co-positivity of HPV 16 and type 18 constituted the least frequent indication (4%; *n* = 17) (Figure 2).

**Figure 2:**
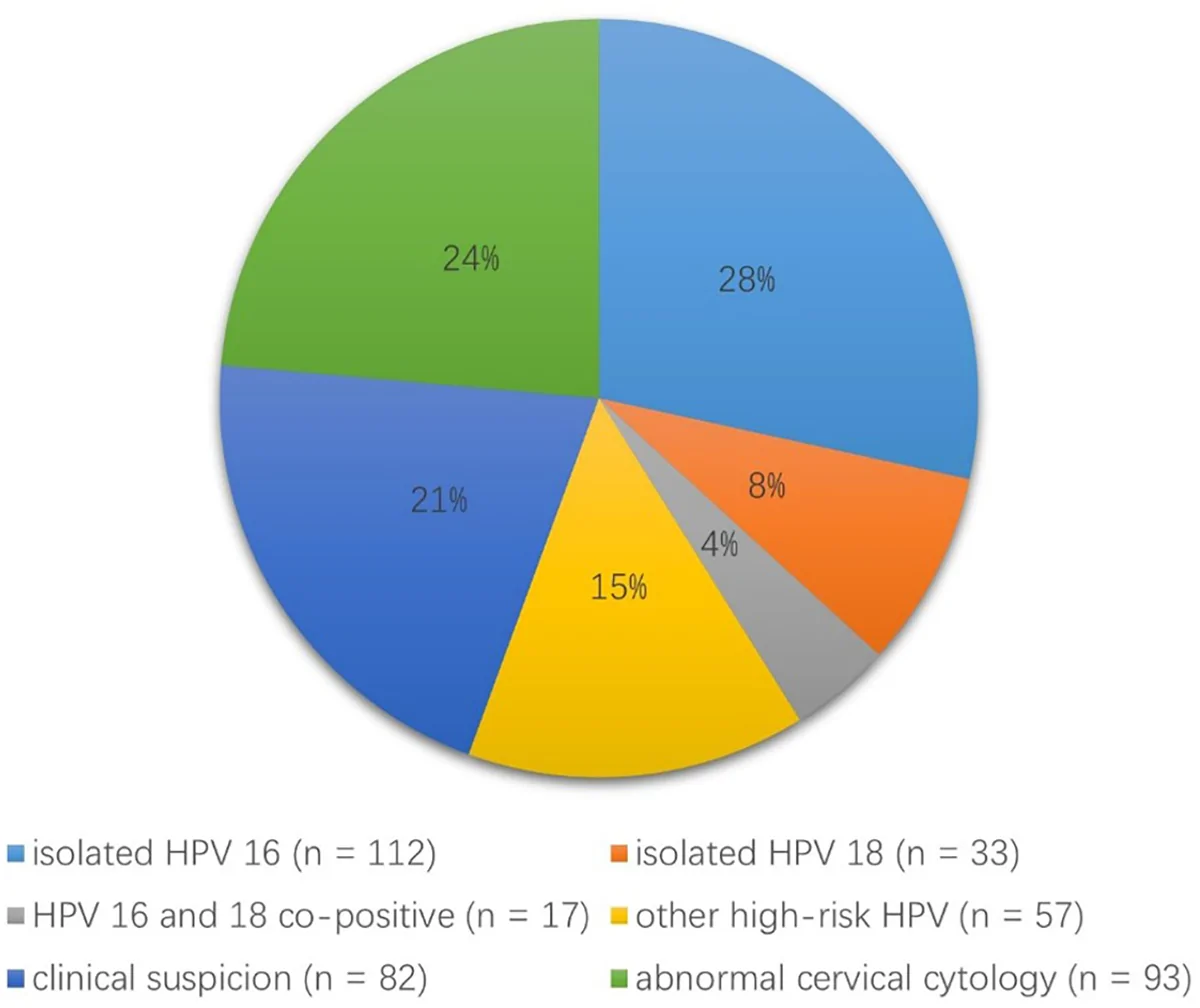
Distribution of indications for colposcopy. HPV, Human Papillomavirus.

### 3.2 Histopathological findings

Histopathological evaluation revealed that cervical dysplasia was absent in 47.3% of the study cohort (*n* = 186); CIN I was identified in 34.5% (*n* = 136); and CIN II–III was noted in 17.7% (*n* = 70). Squamous cell carcinoma was diagnosed in two instances (0.5%) (Figure 3). AGC was identified in one patient; however, no cases of adenocarcinoma *in situ* (AIS) or invasive adenocarcinoma were detected in the cohort. Both invasive malignancies identified were squamous cell carcinomas.

**Figure 3:**
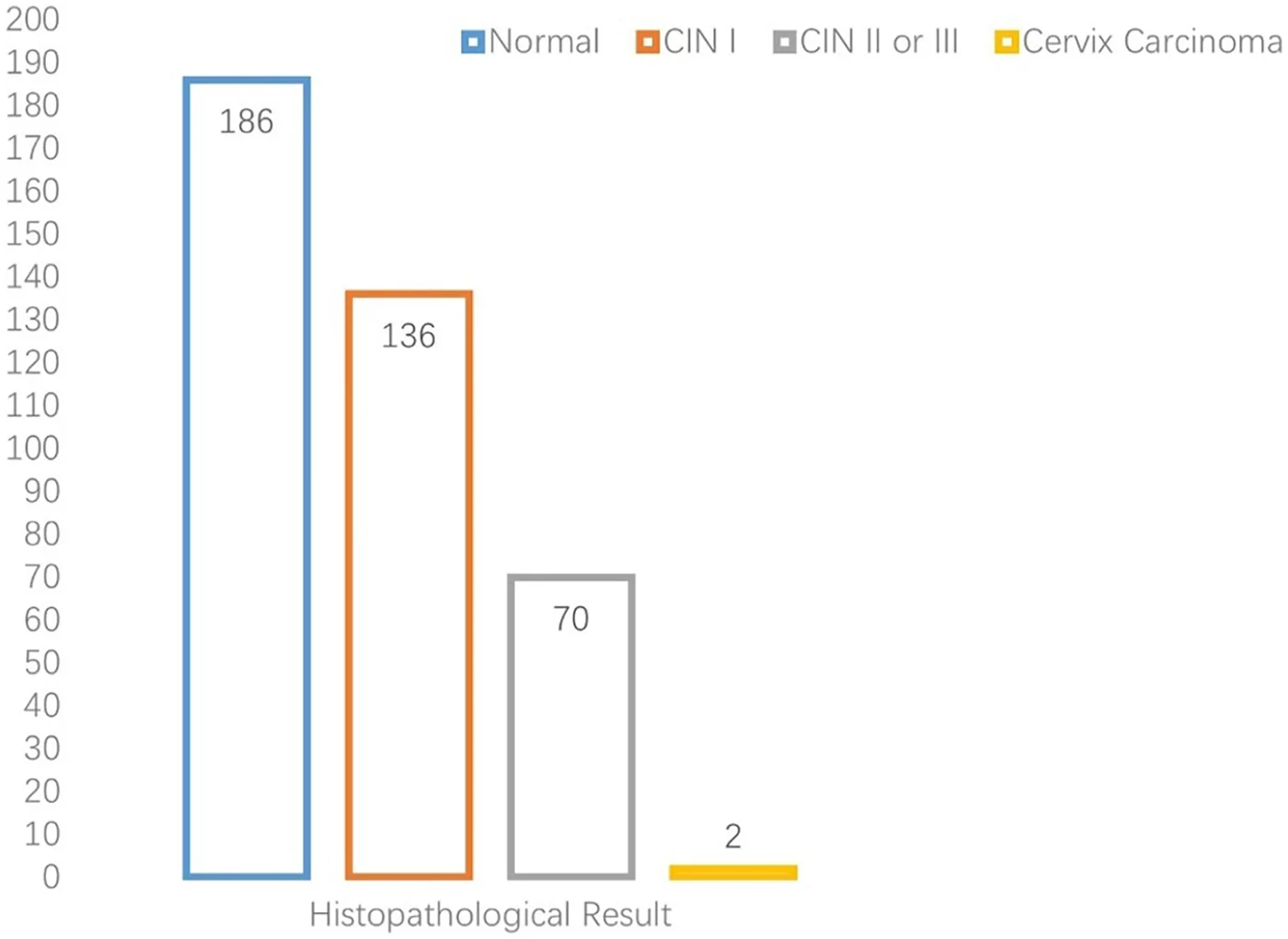
Distribution of histopathological results. CIN, cervical intraepithelial neoplasia.

Among the cohort of cervical dysplasia cases (*n* = 208), isolated HPV 16 positivity was identified in 30.3% of subjects (*n* = 63).

Within the patient group exhibiting CIN I (*n* = 136), clinical suspicion (*n* = 31, 22.8%) and abnormal cytology (*n* = 30, 22.1%) constituted the most frequent colposcopy indications, though isolated HPV 16 positivity accounted for a comparable proportion of cases (*n* = 31, 22.8%).

Regarding the CIN II–III cohort (*n* = 70), the predominant indications were isolated HPV 16 positivity (*n* = 31, 44.3%) and abnormal cervical cytology (*n* = 18, 25.7%).

Two cases of malignancy were observed, one of which involved a patient with isolated HPV16 positivity, and the other patient underwent colposcopy due to abnormal cervical cytology (Table 1).

**Table 1:** Distribution of histopathological results according to colposcopy indications.

Indications for colposcopy (*n* = 394)	No dysplasia (*n* = 186) % (*n*)	CIN I (*n* = 136) % (*n*)	CIN II–III (*n* = 70) % (*n*)	Cervical carcinoma (*n* = 2) % (*n*)
Isolated HPV 16 (*n* = 112)	43.7 (49)	27.7 (31)	27.7 (31)	0.9 (1)
Isolated HPV 18 (*n* = 33)	57.6 (19)	36.4 (12)	6.0 (2)	0.0 (0)
HPV 16 and 18 co-positivity (*n* = 17)	29.4 (5)	41.2 (7)	29.4 (5)	0.0 (0)
Other HR-HPV (*n* = 57)	47.4 (27)	43.8 (25)	8.8 (5)	0.0 (0)
Clinical suspicion (*n* = 82)	51.2 (42)	37.8 (31)	11.0 (9)	0.0 (0)
Abnormal cervical cytology (*n* = 93)	47.3 (44)	32.2 (30)	19.4 (18)	1.1 (1)

HPV, Human Papillomavirus; CIN, cervical intraepithelial neoplasia; HR, high-risk.

### 3.3 Distribution of dysplasia by HPV status and indication

Analysis of the isolated HPV 16-positive cohort revealed that no cervical dysplasia was detected in 43.7% (*n* = 49) of cases; conversely, CIN I, CIN II–III, and malignancy manifested in 27.7% (*n* = 31), 27.7% (*n* = 31), and 0.9% (*n* = 1) of cases, respectively.

Cervical dysplasia was observed in 70.6% (*n* = 12) of patients with HPV 16/18 co-positivity; however, this difference was not statistically significant (*p* = 0.133). Given the small subgroup size (*n* = 17), this finding should be interpreted descriptively (Table 2), whereas its absence was noted in 51.2% of cases with clinical suspicion and 47.3% of those with abnormal cervical cytology.

**Table 2:** Comparison of histopathological results according to colposcopy indications.

Colposcopy indications	Histopathological results					
	Cervical dysplasia		CIN I		CIN II–III	
	(+) (*n* = 208)	(−) (*n* = 186)	(+) (*n* = 136)	(−) (*n* = 258)	(+) (*n* = 70)	(−) (*n* = 324)
	% (*n*)	% (*n*)	% (*n*)	% (*n*)	% (*n*)	% (*n*)
Isolated HPV 16 (+)	56.3 (63)	43.8 (49)	27.7 (31)	72.3 (81)	27.7 (31)	72.3 (81)
Other	51.4 (145)	48.6 (137)	37.2 (105)	62.8 (177)	13.8 (39)	86.2 (243)
Test value	0.751		3.238		10.523	
*P*	0.386*		0.071*		0.001*	
Isolated HPV 18 (+)	42.4 (14)	57.6 (19)	36.4 (12)	63.6 (21)	6.1 (2)	93.9 (31)
Other	53.7 (196)	46.3 (167)	34.3 (124)	65.7 (237)	18.8 (68)	81.2 (293)
Test value	1.553		0.815		3.378	
*p*	0.212*		0.054*		0.066*	
HPV 16 and HPV 18 co-positivity	70.6 (12)	29.4 (5)	41.2 (7)	58.8 (10)	29.4 (5)	70.6 (12)
Other	52 (196)	48 (181)	34.2 (129)	65.8 (248)	17.2 (65)	82.8 (312)
Test value	2.258		0.348		1.649	
*p*	0.133*		0.554*		0.199**	
Other HR-HPV (+)	52.6 (30)	47.4 (27)	43.9 (25)	56.1 (32)	8.8 (5)	91.2 (52)
Other	52.8 (178)	47.2 (159)	32.9 (111)	67.1 (226)	19.3 (65)	80.7 (272)
Test value	0.001		2.573		3.690	
*p*	0.979*		0.108*		0.054*	
Clinical suspicion	48.8 (40)	51.2 (42)	37.8 (31)	62.2 (51)	11.0 (9)	89.0 (73)
Other	53.8 (168)	46.2 (144)	33.7 (105)	66.3 (207)	19.6 (61)	80.4 (251)
Test value	0.668		0.495		3.268	
*p*	0.413*		0.481*		0.070*	
Abnormal cervical cytology	52.1 (49)	47.9 (45)	31.9 (30)	68.1 (64)	19.1 (18)	80.9 (76)
Other	53.0 (159)	47.0 (141)	35.3 (106)	64.7 (194)	17.3 (52)	82.7 (248)
Test value	0.021		0.370		0.161	
*p*	0.882*		0.542*		0.687*	

HPV, Human Papillomavirus; CIN, cervical intraepithelial neoplasia; HR, high-risk.

* Pearson Chi-square test.

** Fisher's Exact test. *p* indicates comparisons between positive (+) and negative (−) groups for each column (Cervical dysplasia, CIN I, CIN II–III).

There was no statistically significant difference among the six indication groups regarding the overall frequency of cervical dysplasia (*p* > 0.05). In isolated HPV 16-positive cases, the detection rate of CIN II–III was 27.7%, and a high proportion of non-dysplastic samples (43.7%) was also observed. The likelihood of CIN II–III was statistically significantly higher in the isolated HPV 16-positive group (*p* < 0.05). No significant association was observed between the degree of dysplasia and colposcopy indications in the other groups (Table 2).

### 3.4 Association with age and smoking status

An analysis of the age distribution showed that the frequency of cervical dysplasia was significantly higher in patients aged ≤44 years (*p* < 0.05). However, no significant difference was observed for CIN (*p* > 0.05). Furthermore, a lower observed prevalence of both overall cervical dysplasia and high-grade cervical dysplasia was found among participants recorded as smokers (*p* < 0.05) (Table 3).

**Table 3:** Distribution of histopathological results according to age and smoking status.

Age and smoking	Histopathological results					
	Cervical dysplasia		CIN I		CIN II–III	
	(+) (*n* = 208)	(−) (*n* = 186)	(+) (*n* = 136)	(−) (*n* = 258)	(+) (*n* = 70)	(−) (*n* = 324)
	% (*n*) [95% CI]	% (*n*) [95% CI]	% (*n*) [95% CI]	% (*n*) [95% CI]	% (*n*) [95% CI]	% (*n*) [95% CI]
Age, yr (mean ± SD: 44.49 ± 10.95)						
≤44	58.7 (122) [53.8–63.6]	41.3 (86) [36.4–46.2]	37.0 (77) [32.2–41.8]	63.0 (131) [58.2–67.8]	20.7 (43) [16.7–24.7]	79.3 (165) [75.3–83.3]
≥45	46.2 (86) [41.3–51.1]	53.8 (100) [48.9–58.7]	31.7 (59) [27.1–36.3]	68.3 (127) [63.7–72.9]	14.5 (27) [11.0–18.0]	85.5 (159) [82.0–89.0]
Test value	6.075		1.219		2.547	
*p*	0.013*		0.269*		0.110*	
Smoking						
Yes	46.6 (110) [41.7–51.5]	53.4 (126) [48.5–58.3]	32.6 (77) [28.0–37.2]	67.4 (159) [62.8–72.0]	13.6 (32) [10.2–17.0]	86.4 (204) [83.0–89.8]
No	62.0 (98) [57.2–66.8]	38.0 (60) [33.2–42.8]	37.3 (59) [32.5–42.1]	62.7 (99) [57.9–67.5]	24.1 (38) [19.9–28.3]	75.9 (120) [71.7–80.1]
Test value	9.023		0.930		7.129	
*P*	0.002*		0.334*		0.007*	

* Pearson Chi-square test. *p* indicates comparisons between positive (+) and negative (−) groups for each column (Cervical dysplasia, CIN I, CIN II–III).

SD, standard deviation; CI, confidence interval; CIN, cervical intraepithelial neoplasia.

### 3.5 Predictors of high-grade cervical dysplasia

Multivariate logistic regression analysis was performed to identify predictors for high-grade dysplasia (CIN II–III), and the results are presented in Table 4. In the unadjusted model, isolated HPV 16 positivity was significantly associated with higher odds of CIN II–III (Model 1: Unadjusted Odds Ratio (UOR) = 2.385, 95% CI: 1.397–4.069, *p* = 0.001). This strong association remained statistically significant after adjusting for age (Model 2: AOR = 2.459, 95% CI: 1.434–4.214, *p* = 0.001), smoking status (Model 3: AOR = 2.318, 95% CI: 1.352–3.977, *p* = 0.002), and both age and smoking status simultaneously (Model 4: AOR = 2.388, 95% CI: 1.387–4.112, *p* = 0.002). No other colposcopy indications showed a significant association with CIN II–III in the adjusted models.

**Table 4:** Multivariate logistic regression analysis of the relationship between colposcopy indications and histopathological results.

Variables	Model 1 (Crude model)		Model 2 (Adjusted model)		Model 3 (Adjusted model)		Model 4 (Adjusted model)	
	UOR (95% CI)	*p*	AOR (95% CI)	*p*	AOR (95% CI)	*p*	AOR (95% CI)	*p*
Cervical dysplasia								
Isolated HPV 16 (+)	0.823 (0.530–1.279)	0.386	1.228 (0.790–1.910)	0.362	1.173 (0.751–1.832)	0.484	1.183 (0.757–1.850)	0.460
Isolated HPV 18 (+)	0.634 (0.309–1.304)	0.216	0.987 (0.969–1.005)	0.149	0.604 (0.291–1.254)	0.176	0.612 (0.295–1.272)	0.189
HPV 16 and HPV 18 co-positivity	2.216 (0.766–6.414)	0.142	0.986 (0.968–1.004)	0.125	2.155 (0.737–6.303)	0.161	2.190 (0.749–6.401)	0.152
Other HR-HPV (+)	0.993 (0.566–1.741)	0.979	0.986 (0.969–1.004)	0.138	1.003 (0.568–1.772)	0.991	1.009 (0.571–1.782)	0.976
Clinical suspicion	0.816 (0.502–1.328)	0.414	0.986 (0.968–1.004)	0.130	0.868 (0.530–1.422)	0.575	0.857 (0.523–1.406)	0.541
Abnormal cervical cytology	0.966 (0.607–1.536)	0.882	0.986 (0.968–1.004)	0.135	0.971 (0.607–1.552)	0.901	0.959 (0.598–1.537)	0.862
CIN I								
Isolated HPV 16 (+)	1.550 (0.960–2.503)	0.073	0.646 (0.400–1.043)	0.074	0.635 (0.392–1.027)	0.064	0.634 (0.392–1.026)	0.064
Isolated HPV 18 (+)	1.092 (0.520–2.293)	0.816	0.998 (0.979–1.017)	0.847	1.079 (0.513–2.269)	0.841	1.079 (0.513–2.271)	0.841
HPV 16 and HPV 18 co-positivity	1.346 (0.501–3.618)	0.556	0.998 (0.979–1.017)	0.840	1.327 (0.493–3.573)	0.576	1.327 (0.492–3.577)	0.576
Other HR-HPV (+)	1.591 (0.899–2.814)	0.111	0.998 (0.979–1.017)	0.824	1.599 (0.903–2.830)	0.107	1.599 (0.903–2.831)	0.107
Clinical suspicion	1.198 (0.724–1.984)	0.482	0.998 (0.980–1.018)	0.872	1.228 (0.739–2.039)	0.428	1.229 (0.739–2.042)	0.427
Abnormal cervical cytology	0.858 (0.523–1.406)	0.543	0.998 (0.979–1.017)	0.826	0.859 (0.524–1.409)	0.548	0.859 (0.523–1.410)	0.547
CIN II–III								
Isolated HPV 16 (+)	2.385 (1.397–4.069)	0.001	2.459 (1.434–4.214)	0.001	2.318 (1.352–3.977)	0.002	2.388 (1.387–4.112)	0.002
Isolated HPV 18 (+)	0.278 (0.065–1.190)	0.084	0.287 (0.067–1.229)	0.092	0.262 (0.061–1.129)	0.072	0.270 (0.063–1.163)	0.079
HPV 16 and HPV 18 co-positivity	2.000 (0.681–5.871)	0.207	0.979 (0.956–1.003)	0.089	1.927 (0.648–5.734)	0.238	2.011 (0.675–5.992)	0.210
Other HR-HPV (+)	0.402 (0.155–1.047)	0.062	0.981 (0.958–1.004)	0.110	0.402 (0.154–1.053)	0.064	0.407 (0.155–1.067)	0.067
Clinical suspicion	0.507 (0.240–1.071)	0.075	0.979 (0.955–1.003)	0.084	0.541 (0.255–1.149)	0.110	0.530 (0.249–1.127)	0.099
Abnormal cervical cytology	1.130 (0.623–2.047)	0.688	0.980 (0.957–1.004)	0.105	1.140 (0.626–2.078)	0.668	1.096 (0.597–2.011)	0.767

Model 1, unadjusted; Model 2, adjusted for age; Model 3, adjusted for smoking status; Model 4, adjusted for age and smoking status. Notably, isolated HPV 16 was not associated with overall cervical dysplasia but specifically with CIN II–III, suggesting it influences severity rather than frequency.

HPV, Human Papillomavirus; CIN, cervical intraepithelial neoplasia; UOR, unadjusted odds ratio; AOR, adjusted odds ratio; CI, confidence interval; HR, high-risk.

## 4 Discussion

We retrospectively evaluated 394 patients who underwent colposcopy in the gynecology clinic of Bandırma Onyedi Eylül University Faculty of Medicine Hospital in the Marmara region between June 2022 and January 2025 to examine relationships among major risk factors for cervical dysplasia, colposcopy indications, and histopathology. Overall, our findings are consistent with national and international literature and provide insights into clinical practice.

### 4.1 Relationship between HPV type and cervical dysplasia

Isolated HPV 16 positivity (Group 1; *n* = 112) was the most frequent indication and was associated with a high burden of dysplasia (56.2%), with CIN I and CIN II–III each observed in 27.7% of cases, closely mirroring rates reported by Tokalıoglu et al. (4). Other Turkish cohorts likewise show substantial dysplasia among HPV 16 positive patients (e.g., CIN I 46.9% and CIN II–III 36.1%) (5).

Our results indicated that HPV16 was present in 22.8% of CIN I and 44.3% of CIN II–III cases, comparable to Kaleli et al. (6) (19.8% and 33.3%, respectively). This demonstrates the increasing prevalence of HPV 16 in higher-grade lesions, a finding consistent with international data, where studies in China and Poland reported HPV 16 positivity in 59.3% and 54.2% of CIN II–III patients, respectively (7, 8), and a study in Israeli women found HPV 16 or 18 positivity in 48.2% of CIN II–III cases (9).

Notably, we also observed one invasive cancer in the HPV 16 group, consistent with HPV 16's established oncogenicity and prior Turkish data (≈1% invasive cancer among HPV 16–positive patients) (4, 10). Collectively, these data reinforce HPV 16 as the predominant driver of precancerous pathology and a key determinant of progression risk, even in CIN I (11). Our results, therefore, support guidelines designating isolated HPV 16 positivity as a high-priority finding warranting prompt colposcopy and close, individualized surveillance, irrespective of cytology.

In contrast, isolated HPV 18 positivity (Group 2) was less frequent and exhibited lower high-grade risk for CIN I and CIN II–III, i.e., 42.4%, 36.4% and 6.1%, respectively. These results indicate that HPV 18 carries a lower risk of cervical dysplasia and especially high-grade lesion development compared to HPV 16. In the literature, it is reported that HPV 18 is more associated with the development of glandular lesions and adenocarcinoma, but not as dominant as HPV 16 in terms of squamous intraepithelial lesions (12). In the study of Dai et al. (13), the rate of CIN I was reported as 36.74% and the rate of CIN II–III was 3.06% in HPV 18-positive cases. These rates are quite compatible with the data in our study. Similarly, in the study of Yüksel et al. (5), the rate of CIN II–III in isolated HPV 18-positive patients was reported as 20.5%, but it was emphasised that this rate was significantly lower compared to HPV 16. Isolated HPV 18 positivity was associated with a lower observed rate of CIN II–III than isolated HPV 16 in this cohort. However, given the limited number of patients in this subgroup, these findings should be interpreted as descriptive and should not be used to support genotype-specific clinical recommendations. Nevertheless, given the established association of HPV 18 with glandular lesions, careful assessment of the endocervical and glandular components remains important in histopathological evaluation.

For dual HPV 16/18 infection (Group 3), both dysplasia (70.6%) and high-grade lesions (29.4%) were frequent, and these rates were higher than those in other groups. Remarkably, both low- and high-grade lesions appeared more common in this group; however, statistical significance could not be demonstrated due to the limited sample size. In the literature, such cases are generally classified as “multiple HR-HPV infections”, and it remains controversial whether this condition poses a higher risk of dysplasia compared with monotype infection. Some studies have reported that multiple HR-HPV infections increase the risk of CIN compared with single-type infections (14, 15). In particular, co-infection with HPV 16 and 18 has been suggested to enhance oncogenic potential through synergistic effects. In a prospective cohort study by Trottier et al. (14), it was shown that the risk of developing CIN II–III was significantly higher in women with multiple HR-HPV infections compared to those with a single type of infection. Similarly, Varesano et al. (15) reported that the presence of multiple HPV types increased the risk of high-grade lesions, especially in combinations including HPV 16. However, in a single-center study, multiple HPV infections were not found to be associated with a significantly higher risk (16). However, subgroup analyses of cases in which HPV 16 and 18 are positive together are limited in the literature, and it is difficult to reach definite results due to the small number of cases.

In our study, the HPV 16/18 co-positive subgroup had a cervical dysplasia rate of 70.6% and a CIN II–III rate of 29.4%. However, this subgroup comprised only 17 patients, and the observed differences were not statistically significant. Therefore, these findings should be regarded as descriptive and exploratory rather than as evidence of an increased clinical risk. The limited sample size and low statistical power preclude firm conclusions regarding the oncogenic impact of HPV 16/18 co-infection. Larger prospective cohorts are needed to determine whether dual HPV 16/18 infection confers additional risk beyond that associated with isolated HPV 16 positivity.

In patients positive for HR-HPV types other than HPV 16 or 18 (Group 4), cervical dysplasia was observed in 52.6% of cases, with CIN I and CIN II–III rates of 43.9% and 8.8%, respectively. These rates indicate an intermediate risk profile compared to HPV 16 and 18. Previous studies have emphasized that other HR-HPV types (e.g., HPV 31, 33, 45, 52, 58) also pose a significant risk for cervical dysplasia and cancer development, though lower than that of HPV 16 (17, 18). In another study, the CIN I and CIN II–III rates were 34.91% and 11.32%, respectively, for other HR-HPV types (13). In our study, corresponding rates were 43.9% and 8.8%, aligning with the literature for low-grade lesions but slightly lower for high-grade lesions. This difference may reflect variations in the study population or screening strategies. It is also recognized that high-risk types other than HPV 16 and 18 may be observed more frequently in some geographical regions, and the specific oncogenic potential of these types may differ (17). Recent hospital-based studies (18, 19) have further elucidated the risk contributions of specific non-16/18 high-risk types, particularly HPV 31, 33, 52, and 58. Other HR-HPV genotypes also demonstrated variable rates of cervical dysplasia; however, because these genotypes were analyzed as a heterogeneous and relatively small subgroup, the findings should be considered descriptive and exploratory. No genotype-specific clinical inference can be made from these data.

### 4.2 Clinical suspicion and abnormal cervical cytology

In patients who underwent colposcopy due to clinical suspicion (Group 5), such as cervical ectropion, abnormal cervical appearance, contact bleeding, and atypical discharge, the rate of cervical dysplasia was 48.8%, the rate of CIN I was 37.8%, and the rate of high-grade lesions (CIN II–III) was 11%. No case of invasive cervical cancer was found in this group. The literature emphasised that HPV positivity in women with suspicious clinical findings is an important indication for colposcopic evaluation (18, 20). Even in cases with normal cytology results, low- and high-grade lesions may be detected if colposcopy is performed due to persistent clinical suspicion (21). Previous studies have reported rates of low-grade lesions between 30% and 50% and of high-grade lesions between 5% and 15% in patients undergoing colposcopy only for clinical suspicion (4, 5). The rates of low- and high-grade lesions found in our study are compatible with these data. In addition, the fact that invasive cancer was not detected in this group confirms that the rates of invasive cancer in patients who underwent colposcopy with clinical suspicion are quite low, similar to the literature (12).

The findings obtained in our study show that the presence of clinical suspicion in gynaecological examination may be an important indication for colposcopic evaluation, independent of cytology and HPV. The fact that a significant proportion of low- and high-grade lesions were found in colposcopies performed with clinical suspicion reveals that this approach has diagnostic value. In conclusion, clinical evaluation based not only on screening tests but also on examination findings has an important place in the early diagnosis of cervical pathologies. Colposcopy, especially in the presence of clinical suspicion, is important for the early detection of progressive lesions and to prevent delay in patient management.

In patients who underwent colposcopy due to abnormal cervical cytology (Group 6), the rates of cervical dysplasia, CIN I, and CIN II–III were 52.7%, 32.3%, and 19.4%, respectively. Dysplasia was not detected in approximately half of the patients (47.3%). These results are broadly consistent with the literature reporting CIN I rates of 30%–40% and CIN II–III rates of 10%–20% in similar cohorts (20, 22). In our study, the rate of high-grade lesions (CIN II–III) was found to be 19.1%, indicating that cytological screening is especially effective in detecting high-grade lesions. However, the fact that dysplasia was not detected histopathologically in almost half of the patients points to the limited specificity of cytological tests. Furthermore, the total number of patients with CIN I and CIN II–III reached 51%, indicating that abnormal cytology is an important warning sign. Our findings are in line with the national and international literature and indicate that colposcopic evaluations based only on abnormal cervical cytology results are of critical importance in the early diagnosis and treatment of high-grade lesions.

In patients who underwent colposcopy with abnormal cervical cytology alone, high-grade lesions (CIN II–III) were detected in approximately one in five patients, while low-grade lesions (CIN I) were detected in one in three. These findings support the effectiveness of current screening programmes and demonstrate that abnormal cytology is a reliable indicator for the early diagnosis of cervical precancerous lesions.

### 4.3 Clinical indications and pathology results

In our study, cervical dysplasia was not detected in approximately half of the patients (47.3%). This rate is comparable to the rates reported in similar patient groups (23). The high rate of non-dysplastic outcomes, particularly in patients undergoing colposcopy due to abnormal cytology or clinical suspicion, likely stems from the inherent subjectivity of clinical examinations and the relatively low specificity of conventional cytological screening, which can lead to over-referrals. The use of colposcopy as a screening method to detect suspicious lesions with high sensitivity is important in terms of preventing unnecessary interventions. However, the fact that dysplasia is not detected as a result of biopsy with colposcopy indication highlights the necessity of developing more personalised approaches in patient management. From a healthcare-resource perspective, the high proportion of non-dysplastic biopsies may create a substantial burden on tertiary colposcopy services. Unnecessary referrals may increase appointment waiting times, procedural costs, pathology workload, and patient anxiety, particularly in regional referral centers serving large populations. More refined secondary triage strategies, including extended HPV genotyping, cytology-based risk assessment, p16/Ki-67 dual staining, or other validated molecular biomarkers, may help improve risk stratification and prioritize colposcopy for women at highest risk of CIN II–III.

Cervical dysplasia rates were similarly high in patients who underwent colposcopy due to clinical suspicion or abnormal cervical cytology, with no significant difference in the incidence of high-grade dysplasia between these groups. This finding supports the diagnostic value of both cytological and clinical assessments, while suggesting that HPV type–specific risk may play a more decisive role in guiding clinical management.

### 4.4 Age and smoking risk factors

In our study, the risk of cervical dysplasia was significantly higher in patients aged ≤44 years compared to patients aged ≥45 years. However, no statistically significant difference was found between age groups in terms of CIN grades. In the literature, age has been frequently analyzed as an important factor in the development of CIN. Our findings are partially consistent with studies reporting an increased risk for CIN development in the young age group due to the high incidence of HPV infection (24).

Conversely, the lack of a statistically significant increase in CIN II–III among the older patients in our study diverges from certain literature. Previous studies report that low-grade lesions tend to decrease with age, while high-grade lesions may persist into advanced ages if left untreated (25). It is suggested that factors such as the decreased regenerative capacity of cervical cells in elderly individuals, the longer persistence of HPV infections, and postmenopausal hormonal changes that affect cervical epithelial structure may facilitate the development of high-grade CIN (26). This discrepancy in our study might be attributed to the demographic characteristics of our study population, the relatively small sample size, or regional factors.

In conclusion, while our findings are partially consistent with the literature, indicating that young age is a risk factor for CIN development, they also present a diverging result, particularly regarding the distribution of CIN II–III in the older age group. This suggests that while the importance of age-specific screening strategies in CIN management remains, the effect of age on CIN grades may not be the same in every population and may vary according to regional demographic characteristics. Therefore, while the hypothesis that hormonal changes and chronic HPV infections at advanced ages pose a potential risk for high-grade CIN remains valid, the strength of this relationship and its reflections in different age groups require more careful, population-specific investigation.

A key finding of our study was a statistically significantly lower incidence of both cervical dysplasia and high-grade lesions in smokers. This result contradicts the extensive literature that identifies smoking as a significant risk factor for cervical dysplasia and cancer (27, 28). We interpret this inconsistency as a result of methodological limitations rather than a true biological effect.

The most likely explanation is misclassification bias arising from retrospectively collected smoking data based on self-report. Due to social desirability bias in a clinical setting, patients may have underreported their smoking habits. Furthermore, the binary categorization of smoking status (Yes/No) in our records failed to capture crucial dosimetric details such as smoking duration, intensity (pack-years), and the distinction between current and former smokers. Such misclassification may have biased the estimated association and limited the reliability of the observed relationship between smoking and cervical dysplasia. Thus, rather than indicating a protective role of smoking, our results underscore the limitations of using retrospectively obtained, self-reported data. It is crucial to emphasize that while self-reported demographic variables are susceptible to such biases, our primary virological outcomes (HR-HPV genotyping via CFX-PCR) and histopathological diagnoses are derived from objective, strictly standardized laboratory assessments. Accordingly, the core findings regarding HPV type-specific risks remain robust, and the isolated findings related to smoking should be interpreted with caution.

### 4.5 Epidemiological and public health evaluation

In our study, cervical malignancy was detected in two patients who underwent colposcopy. This finding suggests that colposcopy is primarily used to detect early-stage lesions, reflecting the effectiveness of current screening strategies. A significant decrease in the incidence of cervical cancer is expected with the widespread use of the HPV vaccine and strengthening of screening programmes worldwide. Population-based studies from countries such as Australia, Sweden, and England have shown that widespread HPV vaccination and regular screening markedly reduce the prevalence of cervical dysplasia and cervical cancer (29–31).

In Turkey, various initiatives have been implemented in recent years to promote HPV vaccination and cervical cancer screening. However, social awareness and access to the vaccine should be increased. Our study reveals the necessity of more effective utilisation of screening and preventive health services, especially for women who are positive for HR-HPV types.

Evaluating the potential public health impact of prophylactic HPV vaccination requires a systematic comparison between the observed genotype distribution in our cohort and the protective valencies of available vaccines. In our population, isolated HPV 16, isolated HPV 18, and dual HPV 16/18 co-infection collectively accounted for nearly three-quarters (74.0%) of all high-risk HPV infections and over half (54.3%) of high-grade lesions (CIN II–III), underscoring the substantial disease burden targeted by bivalent (HPV 16/18) and quadrivalent (HPV 6/11/16/18) vaccines. Notably, isolated HPV 16 represented the single most prevalent high-risk strain and the primary driver of high-grade dysplasia (AOR = 2.388). Nevertheless, non-16/18 high-risk genotypes (Group 4; comprising types 31, 33, 45, 52, and 58) were present in 14.5% of the overall cohort, representing 26.0% of all HR-HPV infections and contributing to 43.8% of CIN I and 8.8% of CIN II–III diagnoses. The nonavalent vaccine, which incorporates coverage against these additional five oncogenic types, extends protection across virtually all high-risk strains identified in this cohort. These observations closely parallel recent epidemiological findings from both national and international literature (32–34), which emphasize that while first-generation vaccines target the predominant oncogenic drivers, the non-negligible burden of non-16/18 high-risk genotypes provides a compelling clinical rationale for adopting the broader 9-valent vaccine in national immunization programs to optimize population-level protection against cervical neoplasia.

### 4.6 Clinical practice and future perspective

The observation that overall dysplasia rates did not differ significantly among indications, whereas CIN II–III was more frequently observed in patients with isolated HPV 16 positivity, supports the established importance of HPV genotype in risk stratification. However, given the single-center and retrospective nature of the present study, these findings should be interpreted as confirmatory rather than practice-changing. They may contribute to the existing evidence supporting genotype-based risk assessment, but broader clinical or public health recommendations require validation in larger, multicenter prospective cohorts.

### 4.7 Limitations

Several methodological limitations should be considered when interpreting the findings of this study. The most fundamental limitation is the single-centre and retrospective design of our study, which significantly constrains the generalizability of our findings to different populations. The study's retrospective nature posed several challenges. First, data on several potentially relevant confounding factors, including sexual behavior, parity, concomitant sexually transmitted infections (STIs), and socioeconomic status, were unavailable in the retrospective records. In particular, parity could not be incorporated into the multivariable analysis because it was not consistently documented across the cohort. Second, smoking data were extracted from self-reported medical records, which may have introduced potential misclassification bias. This limitation could explain why smoking was not identified as a risk factor in the present investigation, contrary to findings in the literature. Although this non-differential misclassification weakened the expected association between smoking and dysplasia, it does not compromise the reliability of our primary findings regarding the strong, independent predictive value of HPV genotyping on colposcopic outcomes. Finally, the lack of follow-up data prevented the assessment of the lesion progression or regression rates over time.

Additional limitations are related to sample selection and statistical power. The evaluation of “clinical suspicion” by different clinicians may have resulted in inter-observer variability and selection bias. This variability might partly account for the weaker association observed between abnormal smears, high-risk HPV positivity, and dysplasia rates compared with published studies. The limited sample sizes of the isolated HPV 18, HPV 16/18 co-positive, and other HR-HPV subgroups resulted in low statistical power and preclude reliable genotype-specific clinical interpretation. Accordingly, findings for these subgroups should be regarded as descriptive and exploratory. The principal statistically supported genotype-specific finding of this study is the independent association between isolated HPV 16 positivity and CIN II–III.

In summary, these limitations suggest the necessity for future research to validate the observed findings and address existing knowledge gaps. Multicenter, prospective studies with larger and more homogeneous cohorts, incorporating additional behavioral and biological risk factors, may provide stronger evidence and facilitate a refined understanding of HPV-related cervical pathology. Additionally, the dichotomization of the continuous age variable based on the population mean, while clinically practical, may have inadvertently reduced statistical power and potentially obscured more nuanced age-related trends. Therefore, our findings should be interpreted in the context of a single-center cohort with limited power for HPV 18 and co-infection subgroups.

## 5 Conclusion

Our findings demonstrate that isolated HPV 16 positivity is a strong independent predictor of high-grade cervical dysplasia (CIN II–III), even after adjustment for age and smoking status. This finding reinforces the central role of HPV 16 in cervical cancer risk stratification and supports its prioritization in clinical follow-up and colposcopy-based management. Although our results are derived from a single-center retrospective cohort and should therefore be interpreted with appropriate caution, they also highlight the potential value of genotype-specific risk assessment in refining current screening and triage strategies. Larger multicenter prospective studies are warranted to validate these findings and to further define the contribution of other high-risk HPV genotypes to clinically meaningful risk stratification.

## Data Availability

The raw data supporting the conclusions of this article will be made available by the authors, without undue reservation.
